# A new species of *Xylopia* (Annonaceae) from the Amazon Basin, with a review of trunciflorous cauliflory in the genus

**DOI:** 10.3897/phytokeys.277.188891

**Published:** 2026-07-07

**Authors:** Morgan Vaughn, Dekon Harper, Lexi Reiter, Elizabeth Dodds, Nancy A. Murray, Keegan Floyd, Gregory W. Stull, David M. Johnson

**Affiliations:** 1 Department of Biological Sciences, Ohio Wesleyan University, Delaware, OH 43015, USA Department of Botany, National Museum of Natural History, Smithsonian Institution Washington United States of America; 2 2473 Indianola Ave, Columbus, OH 43202, USA Department of Biological Sciences, Ohio Wesleyan University Delaware United States of America; 3 Department of Botany, National Museum of Natural History, Smithsonian Institution, Washington, DC 20560, USA Unaffiliated Columbus United States of America

**Keywords:** Bet-hedging, dispersal, key, Late Miocene, Neotropics, phylogeny, pollinators, *Xylopia* XY-peruviana clade

## Abstract

A new cauliflorous species of the pantropical genus *Xylopia* (Annonaceae) is described from the Amazon Basin. *Xylopia rubrolineata***sp. nov**., is shown to be distinct from *X. ochrantha*, with which it had previously been confused. On the basis of its morphology, *Xylopia rubrolineata* is inferred to belong to a small subclade of *X.* sect. *Xylopia*. A review of cauliflory in the genus indicated that the evolution of trunciflorous cauliflory was limited in time and place, occurring in only nine of ca. 220 species, all in two subclades of *X.* sect. *Xylopia* and confined to the Amazon Basin and Brazilian Atlantic Forest. The distribution of the trait within these subclades suggests multiple gains or losses. Cauliflory in *Xylopia* is hypothesized to be a bet-hedging adaptation, which enhanced reproductive success during a period of rapid change in Late Miocene South America that created a mosaic of fluctuating environments and extinctions. An identification key to the nine known trunciflorous species is provided.

## Introduction

**Figure 1.**
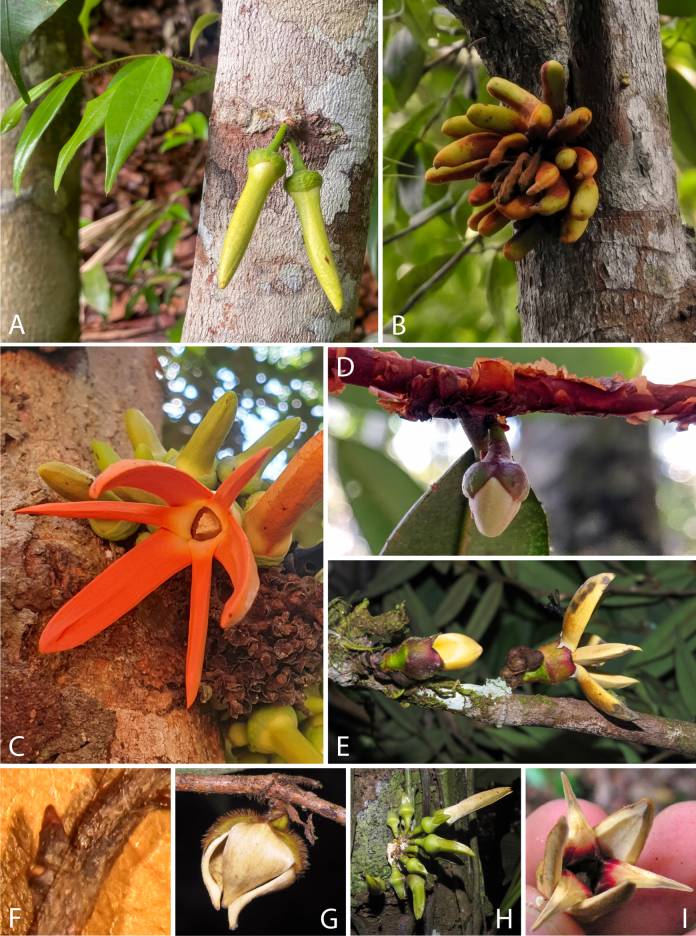
Morphological aspects of cauliflorous Neotropical species of *Xylopia* and of the *Xylopia* XY-peruviana clade. **A**. Twigs and flower buds of *X. benthamii*, Presidente Figueiredo, Amazonas, Brazil, 13 January 2024, E. D. Koch; **B**. Fruit of *X. ochrantha*, Linhares, Espírito Santo, Brazil, 8 October 2022, J. N. F. Rizzo; **C**. Flower of *X. benthamii*, December 2023, Í. Rocha; **D**. Twig of *X. decorticans*, Santa Maria de Jetibá, Espírito Santo, Brazil, 4 October 2023, L. Calazans; **E**. Side view of flower bud and flower of *X. crinita*, Pastaza Canton, Ecuador, 19 September 2022, T. L. P. Couvreur; **F**. Shoot apex of *X. peruviana* R.E. Fr., showing, left to right, the terminal bud scar, pseudoterminal bud, and petiole of leaf, from *Williams 6192* (US), D. M. Johnson; **G**. Flower of *X. longicuspis*, Pastaza Canton, Ecuador, 21 September 2022, T. L. P. Couvreur; **H**. Inflorescence of *X. ulei* Diels, Pastaza Canton, Ecuador, 21 September 2022, T. L. P. Couvreur; **I**. Flower, apical view, of *X. ochrantha*, Pernambuco, Brazil, April 2010, T. Leão. Photographs used under the terms of the Creative Commons license for iNaturalist.

**Figure 2.**
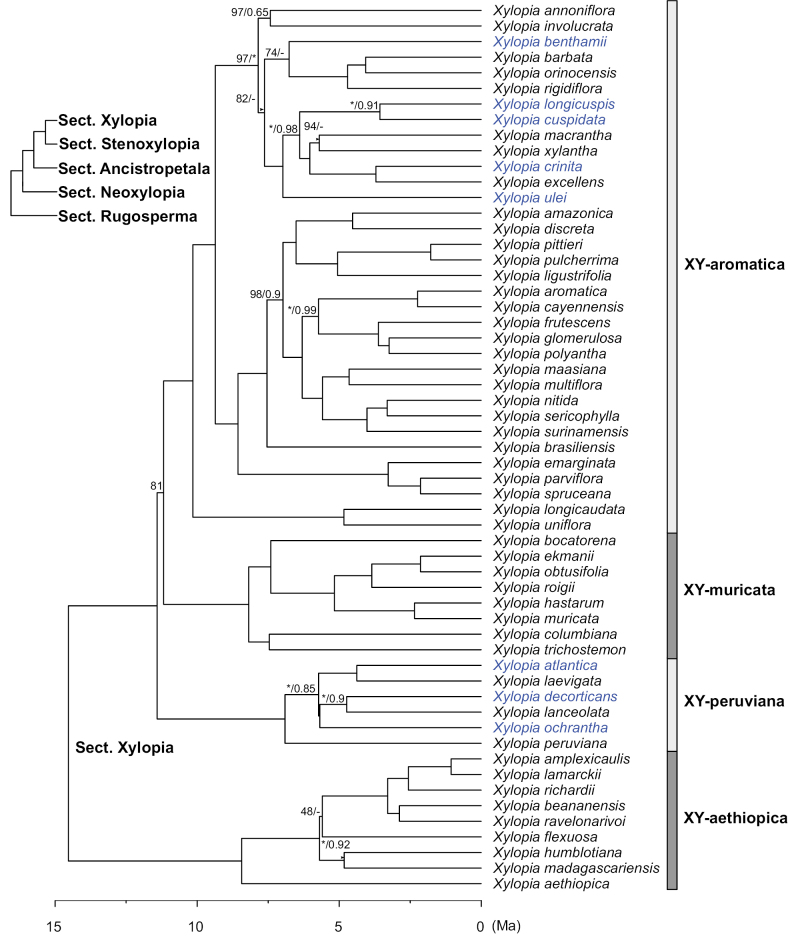
Phylogenetic distribution of trunciflory in *Xylopia* sect. *Xylopia*. Species in which trunciflory has been documented are highlighted in blue. Phylogeny and dates are those determined by Nge et al. (2026): all branches are fully supported based on maximum likelihood (RAxML) and species-tree (ASTRAL) analyses unless otherwise indicated. Submaximal values are displayed as “bootstrap/local posterior probability;” an asterisk indicates full support for the undisplayed value; a hyphen indicates that the particular clade was not recovered in the ASTRAL analysis. Names in the right-hand column are clade names as used in Johnson et al. (2025) and Nge et al. (2026).

*Xylopia* L. (Annonaceae) is a pantropical genus of ca. 220 species of woody flowering plants. The axillary flowers have valvate, often narrowly oblong petals (Fig. 1). The fruits are aggregates of dehiscent monocarps that present the seeds against a brightly colored endocarp, the seeds providing arils or sarcotestas as fleshy rewards for dispersers (Johnson et al. 2025). In the Neotropics, ca. 57 species are currently recognized, over half occurring in the Amazon River Basin, where species new to science continue to be described (Bagstad and Johnson 1999; Pontes-Pires et al. 2021a, 2021b). A recent molecular phylogeny established the monophyly of the Neotropical species, dating their arrival at ca. 11.6 Ma, and recovered three clades, XY-peruviana, XY-muricata, and XY-aromatica (Johnson et al. 2025; Nge et al. 2026; Fig. 2).

The first aim of this study is to assess the taxonomic status of a group of *Xylopia* collections from Amazonian Bolivia and Brazil that appeared, on morphological grounds, to belong to the XY-peruviana clade (see Appendix 1). They resemble and have often been identified as *Xylopia ochrantha* Mart., a species endemic to the Atlantic coastal forests of eastern Brazil (Bazante and Alves 2021). The plants share two unusual traits for the genus, blunt, broadly ovate outer petals (Fries 1930) and cauliflory, as well as elongate monocarps with seeds oblique to the long axis.

Cauliflory occurs in many flowering plant families and is widespread in the Annonaceae (Onstein et al. 2019; Amancio et al. 2026). Mildbraed (1922) distinguished four main subtypes: ramiflory (inflorescences on leafless branches), trunciflory (flowers arising from the trunk bole), basiflory (flowers arising from the base of the trunk), and flagelliflory (flowers on elongate branches arising from the trunk). In *Xylopia*, ramiflory is uncommon (e.g., Johnson and Murray 2015, 2018, 2020), and trunciflory is known only in a small number of South American species in the XY-peruviana and XY-aromatica clades (Lobão and Johnson 2007): *X. benthamii* R.E. Fr., *X. cuspidata* Diels, *X. decorticans* D.M. Johnson & Lobão, *X. longicuspis* R.E. Fr., and *X. ochrantha* (Figs 1, 2). Many misidentifications have occurred among them. The second aim of this study is to identify clear distinctions between the trunciflorous species.

Cauliflory has long attracted attention, beginning with the proposal by Wallace (1878) that it made flowers of trees more visible to pollinators in the tropical forest understory. Richards (1952) and many subsequent authors have confirmed the understory habit of most cauliflorous species. Understory movement of pollen varies widely, with pollen moved, for example, only short distances by small, weak-flying beetles (Grant et al. 2019, 2021) but greater distances by bats (Gottstein et al. 2025). Cauliflory may improve access to floral visitors: Warren et al. (1997) found in two different species twice as many floral visitors to inflorescences on the trunk as to those in the canopy of the same individual, as well as greater fruit set. Finally, flowering and fruiting in cauliflorous plants may not be constrained by the production of new vegetative shoot growth, offering more flowers, longer flowering periods, or other changes that augment reproductive success (Armstrong and Marsh 1997; Harrison et al. 2012).

The effect of cauliflory on seed dispersal has also been examined. In species of *Ficus* L. subgenus *Sycomorus* Raf., fruit placement, independent of fruit color and size, affected the composition of frugivore cohorts (Harrison et al. 2012). In the cauliflorous liana *Marcgravia longifolia* J.F. Macbr. (Marcgraviaceae), more frugivore species were present in the canopy, but these dispersers made fewer visits than understory frugivore species (Thiel et al. 2023). Tongkok et al. (2020) found that cohorts of available dispersers varied with site depending on geography, seed predation, and other factors.

Recent studies indicate that cauliflorous and non-cauliflorous species are often intermixed phylogenetically, e.g., in *Ficus* (Moraceae; Harrison et al. 2012), *Adenocalymma* Mart. ex Meisn. (Bignoniaceae; Fonseca and Lohmann 2017; Fonseca et al. 2026), and *Desmopsis* Saff. (Annonaceae; Martínez-Velarde et al. 2023). A dated phylogenetic reconstruction of *Xylopia* and the identification of possible ecological and historical factors in the diversification of the genus (Johnson et al. 2025; Nge et al. 2026) led to the third aim of this study: to review the ways evolutionary history, habitat, floral biology, and seed dispersal may have shaped cauliflory in *Xylopia* and suggest a hypothesis concerning the evolution of the trait.

## Methods

Collections from the following herbaria were examined directly or in digital images: AAU, F, K, L, MO, NY, OWU, U, and US (acronyms from Thiers 2026, continuously updated). Measurements were taken from dried material. The largest leaves of a collection, up to three where possible, were measured. Smaller measurements were made with a Zeiss stereomicroscope and ocular micrometer to the nearest 0.1 mm, and drawings of small parts were made using a drawing tube. Collection localities were mapped with ArcGIS Pro, which was also used to produce the final map. The map uses WGS 1984 (Geographic Coordinate System) for XY coordinates and EGM96 height (Vertical Coordinate System, Gravity-Related) for Z coordinates and includes the Esri base layers “World Countries,” 5 December 2025 (https://www.arcgis.com/home/item.html?id=ac80670eb213440ea5899bbf92a04998, accessed April 20, 2026), and “Environment Map (World Edition),” 19 March 2026 (https://www.arcgis.com/home/item.html?id=a69f14ea2e784e019f4a4b6835ffd376, accessed 20 April 2026). For the World Countries layer, color was altered from orange to transparent to allow overlays of other data, and outline color was altered from brown to dark gray to provide contrast. For the Environment Map layer, the Environment Detail and Label and Environment Watersheds default layers were omitted for clarity. For specimens lacking coordinate data, coordinates were determined from the locality description given on the label. Specimens from the same locality are represented by a single symbol on the map. iNaturalist (www.iNaturalist.org) records also provided coordinate data for recent observations of the species, phenological data on flowering and fruiting, and color documentation of flowers and fruits, augmenting data from herbarium specimens. Photographers are credited in the figure captions and Acknowledgements. Historical Extent of Occurrence (EOO) and Area of Occupancy (AOO) were calculated (IUCN 2012), the latter using a population area of 4 km^2^ as in previous work on this genus (e.g., Johnson and Murray 2018), along with observations on disturbance and dates of collection to make a preliminary conservation assessment. The presence of cauliflory in *Xylopia* species was documented from herbarium specimens, published taxonomic literature, and iNaturalist observations; data from these sources were used to construct a key to trunciflorous species. For comparison of present-day climate profiles of cauliflorous and non-cauliflorous species of the XY-peruviana clade and XY-aromatica subclade, WorldClim bioclimatic data (Fick and Hijmans 2017) assembled for Johnson et al. (2025) were used.

## Results

### Xylopia rubrolineata

Taxon classificationPlantaeMagnolialesAnnonaceae

D.M.Johnson & N.A.Murray sp. nov.

42EBFCF6-F521-5B81-A82A-D6122E24F14C

urn:lsid:ipni.org:names:77382773-1

**Figure 3.**
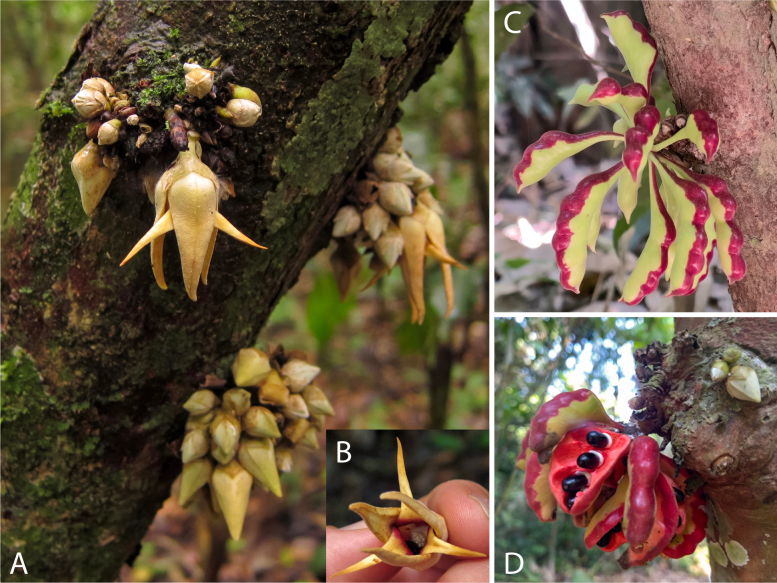
*Xylopia rubrolineata* (XY-peruviana clade). **A**. Cauliflory, Alta Floresta, Mato Grosso, Brazil, 12 October 2011, R. Hoyer; **B**. Flower at anthesis, apical view, Alta Floresta, Mato Grosso, Brazil, 12 October 2011, R. Hoyer; **C**. Fruit on trunk, Alta Floresta, Mato Grosso, Brazil, 19 August 2025, M. Z. Ferreira; **D**. Inflorescence on trunk, showing dehisced monocarp and seeds, and flower buds, Cristalino Lodge, Mato Grosso, Brazil, 12 August 2022, S. Freire. Photographs used under the terms of the Creative Commons license for iNaturalist.

**Figure 4.**
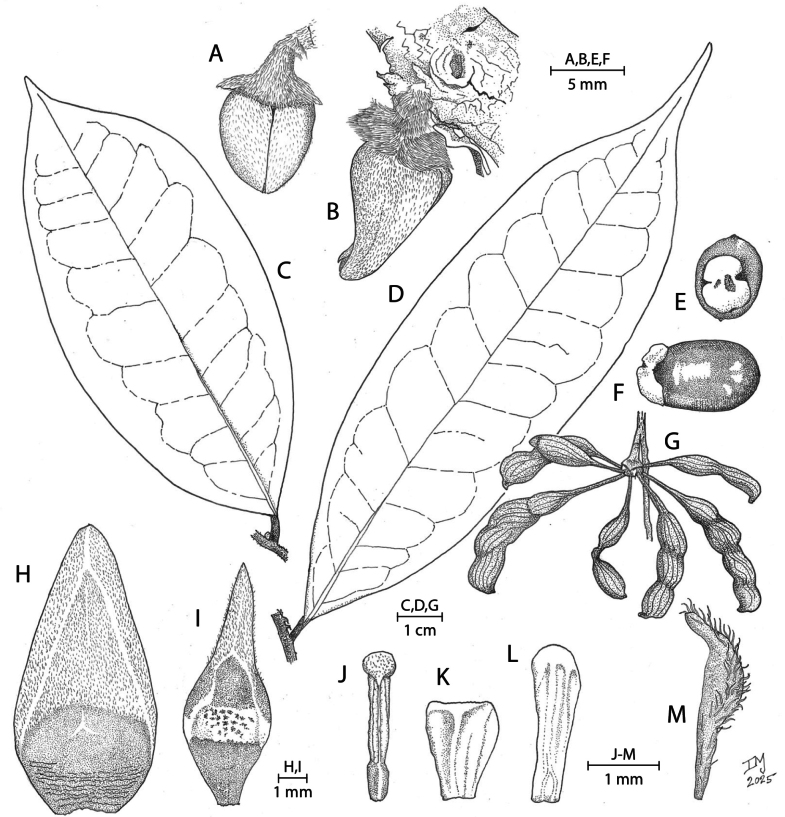
*Xylopia rubrolineata*. **A**. Flower bud; **B**. Flower bud showing cauline attachment; **C, D**. Leaves; **E**. Seed, view of micropylar end; **F**. Seed, side view; **G**. Fruit; **H**. Outer petal, adaxial view; **I**. Inner petal, adaxial view; **J**. Stamen, abaxial view; **K, L**. Staminodes, abaxial view; **M**. Abscised stigma, side view; **A, C**. From *Sperling et al. 5987* (NY); **B, D**. From *Chatrou et al. 410* (OWU); **E–G**. From *Silva & Souza 2334* (OWU); **H, I**. From *Silva & Souza 2459* (OWU); **J–M**. From *Sperling et al. 5987* (MO).

Figs 3, 4, Chatrou et al. (2012, Frontispiece G)

#### Type.

**Bolivia** • Bení, Prov. Vaca Diez, Riberalta, Camino Santa Rosa, road (and trails) leading S from km 13 of Riberalta–Guayaramerin road, 11°02'S, 66°01'W, 19 Nov 1989 (buds), *Daly et al. 6265* (holotype MO, isotypes NY, U [U.1075910]).

#### Diagnosis.

Species resembling *Xylopia ochrantha* Mart. in its cauliflory and short broad yellowish petals, but differing in its chartaceous rather than subcoriaceous leaves, smaller outer petals with more acute apices, and torulose, stipitate, glabrate monocarps, which are pale green with a bright red abaxial stripe *in vivo*; the monocarps of *X. ochrantha*, in contrast, are smooth or only slightly indented between seeds, have shorter and thicker stipes, are densely velutinous, and are greenish and faintly tinged with red *in vivo*.

#### Description.

Shrubs to trees (1–) 3–15 (–25) m tall, dbh to 35 cm, bark peeling. ***Twigs*** reddish gray, brown, or reddish brown, with pseudoterminal resting buds, initially loosely appressed-pubescent, soon glabrate. ***Leaves*** with larger blades 7.1–17.3 cm long, 2.4–5.6 cm wide, chartaceous, concolorous or rarely glaucous abaxially, elliptic or occasionally obovate, base cuneate, apex acuminate, acumen 4–30 mm long, margins flat, glabrous adaxially, pubescent along the midrib but otherwise glabrous abaxially; midrib sunken adaxially, raised abaxially, secondary veins 6–13 per side, diverging from midrib at 50–80°, brochidodromous, secondary and higher-order veins raised, plane or sunken adaxially, plane to raised abaxially; petiole 3–9 mm long, shallowly canaliculate, sparsely pubescent to glabrate. ***Inflorescences*** up to 8-flowered on the trunk, 1–3-flowered on leafless branches, or rarely arising from the axils of leafy twigs, peduncle absent, pedicels 2–4 mm long, 1.5–2.5 mm thick, densely appressed-pubescent with pale orange-brown hairs, bracts 1–2, caducous, 3–5 mm long, ovate to orbicular; buds lanceolate to ovoid, apex obtuse, occasionally acute. ***Sepals*** connate for 0.8–1.5 mm at the base, 3.5–6.0 mm long, 4.0–4.8 mm wide, coriaceous, elliptic to triangular, apex obtuse to acute, densely and coarsely sericeous abaxially. ***Petals*** valvate, brownish yellow to cream-colored *in vivo*, the adaxial bases of the petals purple or maroon, fleshy; outer petals erect at maturity, 9.6–13.0 mm long, 4.5–5.9 mm wide, 0.7–1.3 mm thick, ovate to lanceolate, base concave, apex acute to slightly obtuse, weakly keeled abaxially, appressed-pubescent with pale orange-brown to whitish hairs on both surfaces; inner petals squarrose at anthesis, (5.4–) 6.5–10.1 mm long, 2.7–4.8 mm wide, angular-ovate to angular-lanceolate, base shallowly concave adaxially, apex broadly to narrowly acuminate, acumen 1.8–4.0 mm long, keeled from midpoint to apex adaxially, proximally giving way to a groove that widens into the basal concavity, puberulent on apical half, glabrous on basal half, on both surfaces. ***Stamens*** 100–240, 1.5–2.6 mm long, narrowly oblong, anther connective apex 0.3–0.4 mm long, truncate to slightly dome-shaped, glabrous, anthers indistinctly septate, with 9–11 locules; outer staminodes 1.6–2.2 mm long, oblong, apex obtuse, innermost stamens c. 1.3 mm long but not appearing staminodial; staminal cone 2.1–3.3 mm in diameter, 1.7–2.4 mm high. ***Carpels*** 8–15, ovaries enclosed by staminal cone, 1.7–2 mm long, narrowly oblong, densely pubescent, stigmas 1.6–2.7 mm long, narrowly ellipsoid, hairy. ***Torus*** flat, 2.4–3.9 mm in diameter. ***Fruit*** of up to 15 monocarps borne on a pedicel 3.0–7.5 mm long, 2.8–3.8 mm thick, coarsely pubescent, torus of fruit 4–7 mm in diameter, with remnants of staminal cone often adhering; monocarps light green to yellowish green with a distinct longitudinal red stripe along the abaxial ridge *in vivo* only, 2.0–5.1 cm long (including stipe), 0.7–1.1 cm wide, 0.6–0.8 cm thick, narrowly oblong to oblong, torulose, terete, obliquely wrinkled, initially with fine, weakly appressed hairs but becoming glabrate, base contracted into a stipe 5–12 mm long, 1.2–1.6 mm thick, apex obtuse or sometimes with a short downcurved beak ca. 1 mm long, pericarp 1.0–1.2 mm thick. ***Seeds*** 1–7 per monocarp in a single row, oblique to long axis of monocarp, 7.4–12.8 mm long, 5.4–6.3 mm wide, 3.9–5.5 mm thick, ovoid to ellipsoid, black and shiny (sarcotesta absent), smooth, perichalazal ring indistinct, plane to slightly raised, endosperm ruminations platelike in quadrants; aril white, bilobed, the lobes sometimes overlapping, 2.0–2.5 mm long, 3.0–4.0 mm wide.

#### Phenology.

Specimens were collected with flowers in June and September–December and with full-sized fruits in June–August; in addition, there are iNaturalist photographs of flowers in October and full-sized fruits from February–August from the Alta Floresta/Cristalino area. Collections made in the Serra dos Carajás in June 1982 included both flowers and mature fruits.

#### Etymology.

The species is named for the prominent red stripe along the abaxial surface of the monocarp (Fig. 3C, D).

#### Distribution and habitat.

**Figure 5.**
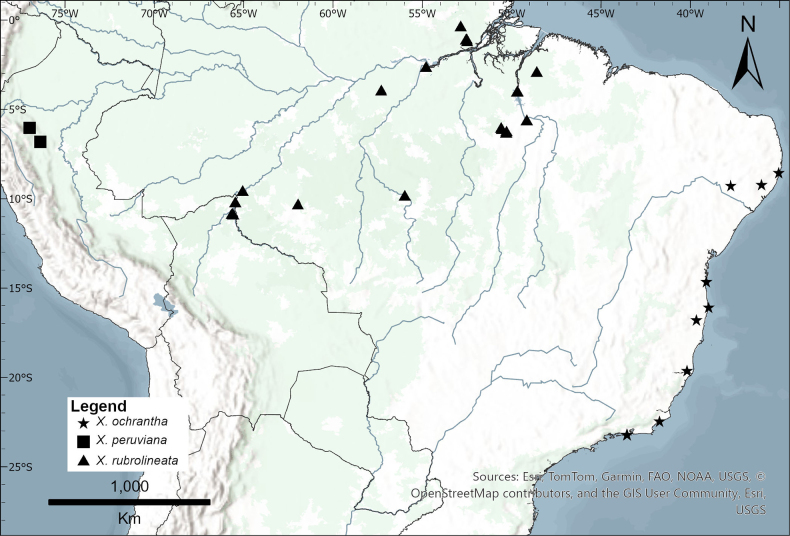
Distributions of *Xylopia ochrantha*, *X. peruviana*, and *X. rubrolineata* in South America. Green shading indicates forest cover as of January 2026. “World Countries” and “Environment Map” layers are shown.

Distributed from northern Bolivia eastward to Pará state, Brazil, primarily south of the Amazon River, but with populations along the Jarí River in northern Pará on the border with Amapá (Fig. 5). Forests on terra firme, sometimes disturbed, on sandy and clay soils, at elevations of 50–650 m; liana forest was mentioned as the habitat at one locality.

#### Local names and uses.

Bita (Chácobo, *Stijfhoorn 700*), isonobichi (Chácobo, *Bèrgeron 273*), peraquina (*Chatrou 410*), tëtëmëtoijoni, a leaf decoction is taken to cure liver problems, and wood is used as fuel (Chácobo, *Boom 4125, 4793*); tëtëmëtsisi, “Use: sweet fruits eaten” (Chácobo, *Boom 4627*); and tëtëmëtsisijoni (Chácobo, *Bèrgeron 273*).

#### Preliminary conservation assessment.

*Xylopia rubrolineata* has an EOO of 1,041,013 km^2^ and an AOO of 128 km^2^. The AOO reflects the fact that the plants are known from only approximately 14 localities in three small areas within the distribution (Fig. 5). Only five collections have been made since 1992, all from the southwestern Amazon, but the presence of the species in the Alta Floresta region has been documented over the period 2 February 2000–19 August 2025. Many of the collections have come from areas influenced by mining, logging, and reservoir construction. The ombrophilous forests in the Alta Floresta region where the species occurs have also been shown to be vulnerable to fire (Rocha et al. 2025). Several collectors noted that the plants were growing in disturbed habitats, but given land use in these areas, these could be residual populations. The EOO places the species at Least Concern, while the AOO places it at Endangered. Given these considerations, the species could be assessed under criterion B as either Near Threatened based on the large EOO or Vulnerable based on the inferred threats and few recent collections (IUCN 2012).

#### Additional specimens examined.

(fl = flower, fr = fruit, st = sterile, yg = young) **Bolivia – Bení** • S. Bèrgeron 273 (L); Vaca Diez, Alto-Ivón; 11°45'S, 66°02'W; alt. 200 m; Aug 1992; fr • B.M. Boom 4125 (NY); Prov. Vaca Diez, vicinity of Chácobo village Alto Ivon; 11°45'S, 66°02'W; alt. ca. 200 m; 8 Dec 1973; bud • B.M. Boom 4627 (NY); same locality; same coordinates; same elevation; same date; st • B.M. Boom 4793 (NY); same locality; same coordinates; same elevation; 27 Mar 1984; ygfr • E. Stijfhoorn 700 (NY); same locality; same coordinates; same elevation; 1 Jul 1991; fr • L.W. Chatrou et al. 364 (OWU); Prov. Vaca Diez, San Pablo, 45 km NE of Riberalta along road to Cachuela Esperanza; 10.37°S, 65.46°W; alt. 250 m; 2 Nov 2001; st • L.W. Chatrou et al. 410 (OWU, U—2 sheets); Prov. Vaca Diez, Santa Maria, 5 km SE of Riberalta; 10.59°S, 66.06°W; alt. 220 m; 8 Nov 2001; fl – **Pando** • G.T. Prance et al. 5767 (NY); W bank of Rio Madeira opposite Abunã; 9 Jul 1968; fr**Brazil – Amazônas** • J.L. Zarucchi et al. 3101 (INPA n. v. [A. Pontes-Pires pers. comm.], MO, NY, U); Mun. de Maués, basin of Rio Maués, Rio Urupadi at mouth of Igarapé Quininha; 3°45'S, 57°17'W; 19 Jul 1983; fr – **Mato Grosso** • G.S. Henicka 133 (K); Mpio de Novo Mundo, Parque Estadual Cristalino; 9°37'50"S, 55°55'15"W; alt. 257 m; 16 Sep 2007; fl • Projeto Flora Cristalino [D. Sasaki et al. collectors] 225 (K000447378); Município de Novo Mundo, área particular de preservação ambiental, delimitada pelo Parque Estadual Cristalino e pelos Rios Teles Pires e Cristalino, nas proximadades de sua confluência, a leste do Rio Cristalino; 9°37'50"S, 55°55'15"W; 19 Jul 2006; fr – **Pará** • C.C. Berg et al. BG 627 (MO, NY, U); Serra dos Carajás, Serra do Norte, ca. 20 km N of AMZA Exploration Camp; ca. 6°S, 50°15'W; 18 Oct 1977; fl • C.C. Berg et al. BG 632 (NY, U); same locality; same coordinates; same date; fl • D.C. Daly et al. D832 (MO, NY, U, US); Fazenda Borba Gato, near Rio Acará, 30 km W of Tomé-Açu-Paragominas road, leave road approx. 30 km S of Tomé-Açu, Municipio Acará; approx. 2°40'S, 48°35'W; 5 Nov 1980; fl buds • D.C. Daly et al. 1152 (NY–2 sheets, U, US); approx. 18 km from Tucuruí, 16 km S on old BR 422, then 2 km W on overgrown logging road; approx. 3°53'S, 49°41'W; 1 Nov 1981; fl buds • D.C. Daly et al. 1172 (NY, U); same locality; same coordinates; same date; fl buds • U.N. Maciel et al. 780 (NY); Marabá, Carajás, Serra Norte, estrada do N-1, 29 km do acampamento; 7 Aug 1982; fl buds • F.E. Miranda et al. 382 (NY); Tucuruí; 1 Sep 1983; fl buds • E. de Oliveira 3504 (NY); Rio Jarí, estrada que liga Monte Dourado a Caracurú; 11 Nov 1967; fr • E. de Oliveira 3540 (NY); Rio Jarí, Serra de Monte Dourado, à 3 km da margem; alt. 140 m; 14 Nov 1967; fl • M.J. Pires 1789 (MO); Almeirim, Monte Dourado, gleba Monte Dourado da Reserva Genetica do Jari; −52.55, −0.85; 8 Oct 1987; fr • J. Ramos 1077 (NY); Tucuruí, próximo ao lago Cagancho; 14 Nov 1983; fl buds • J. Revilla et al. 8563 (NY); Tucuruí, PA-149; 5 Oct 1983; fl buds • N.A. Rosa et al. 4050 (NY); rodovia Tucurui, km 26, Repartimento; 6 Apr 1981; fl buds • M.R. Santos 495 (NY, U); munic. de Almeirim, Monte Dourado, Beiradão, estrada da caixa d’agua; 6 Dec 1978; fr • R.S. Secco et al. 393 (MO, U); Marabá, clareira à direita da estrada para o acampamento azul; 29 May 1982; fr • A.S. Silva et al. AS 10 (AAU, NY, U); BR 230, Transamazon Highway, estrada N-1, municipio de Marabá; 15 Oct 1977; fl • M. Silva & R. Souza 2271 (U); Santarém, km 35 da estrada do Palhão, ramal para o Igarapé do Pilão; 16 Aug 1969; fr • M. Silva & R. Souza 2334 (OWU); same locality; 20 Aug 1969; fr • M. Silva & R. Souza 2375 (MG n. v. [A. Pontes-Pires, pers. comm.], OWU–2 sheets); Santarém, km 35 da estrada do Palhão, arredores do Acampamento do Igarapé Curupira; 24 Aug 1969; fr • M. Silva & R. Souza 2459 (OWU); same locality; 1 Sep 1969; fl • N.T. Silva 1616 (NY); região do Rio Jarí, Rio Jarí, Monte Dourado, Bandeira, Planalto C; 13 Jan 1969; fl • N.T. Silva 1695 (NY, OWU); região do Rio Jarí, estrada entre Braço e Planalto A; 28 Jan 1969; fl • N.T. Silva 2402 (NY, U); Jari, estrada entre Planalto A e Tinguelim, km 16; 12 Jul 1969; ygfr • N.T. Silva 2404 (NY, U); same locality; same date; ygfr • C.R. Sperling et al. 5984 (MO, NY, U, US); Serra dos Carajás, AMZA camp 3-Alfa; 5°48'S, 50°33'W; alt. ca. 300–475 m; 8 Jun 1982; fl buds • C.R. Sperling et al. 5985 (NY, U, US); same locality; same coordinates; same elevation; same date; fr • C.R. Sperling et al. 5987 (MO, NY, U, US); same locality; same coordinates; same elevation; same date; fl buds • C.R. Sperling et al. 6089 (NY, U, US); Serra dos Carajás, ca. 2 km NW of Rio Itacaiúnas ferry crossing on road to AMZA camp Saloro-2, 3-Alfa; 5°53'S, 50°30'W; alt. ca. 200 m; 11 Jun 1982; fr • C.R. Sperling et al. 6243 (NY, U, US); Serra dos Carajás, 10 km E of AMZA camp N-1 and 5–10 km along the entrance road to AZUL, an abandoned manganese exploration camp; 6°05'S, 50°16'W; alt. 600–650 m; 19 Jun 1982; fr – **Rondônia** • G.T. Prance et al. 5646 (INPA n.v. [A. Pontes-Pires pers. comm.], NY, U); basin of Rio Madeira, road to Cassiterite mines in Serra dos Tres Irmãos, north bank of Rio Madeira, 8 km above Mutumparana; 5 Jul 1968; fr • P.H. Labiak et al. 6263 (NY); Ji-Paraná, Reserva Biológica do Jaru, trilha atrás do alojamento de Pesquisadores; 10°08'42"S, 61°54'25"W; alt. 118 m; 9 Jun 2015; fr.

#### Notes.

The petals of *X. rubrolineata* are thinner and approximately half as long as those of *X. ochrantha*, and the leaves are chartaceous rather than subcoriaceous. The monocarps of *X. rubrolineata* are consistently torulose, with a long, slender stipe (5–12 mm long, 1.2–1.6 mm thick) and fine, sparse indument, while those of *X. ochrantha* have at most faint indentations between the seeds, shorter stipes (4–8 mm long, 2.5–3 mm thick), and coarse, dense pubescence. *Xylopia rubrolineata* differs from *X. peruviana* in cauliflory, the longer and more gradual acumen of the leaf, again the smaller petals, and the blunt rather than sharply acute apex of the glabrous monocarps. From both species, *X. rubrolineata* consistently differs in the pronounced abaxial red stripe on the monocarps, which has attracted the attention of many photographers (Fig. 3C, D). Generally, fruit color in *Xylopia* is not helpful for identification, changing as the monocarps mature, but in this case, the red stripe is present up to the final maturation of the fruit, as marked by dehiscence of the monocarps (Fig. 3D). The shoot tips of *X. rubrolineata* and all species of XY-peruviana were found to have a pseudoterminal vegetative bud covered by bud scales; the subtending leaf usually remains attached (Fig. 1F).

*Xylopia egleriana* Aristeg. ex Maas, known from one incomplete collection from the Jarí River basin of Pará, Brazil (Maas et al. 1993), was examined because it overlaps in distribution with *X. rubrolineata*. *Xylopia egleriana*, however, has shorter, thicker leaves rounded at the base, adaxially sunken secondary veins, distinctly connate sepals, and coarse ferruginous hairs on the floral parts. Maas et al. (1993) suggested a relationship of *X. egleriana* to *X. cuspidata* and similar species, but it was not sequenced for molecular phylogenetic placement in the most recent study of the genus (Nge et al. 2026).

### Cauliflory in *Xylopia*

Trunciflory is present in nine *Xylopia* species, four from the XY-peruviana clade and five from a subclade of XY-aromatica (Table 1). Cauliflory is fixed within species and includes both ramiflory and trunciflory, except in *X. benthamii* and *X. ulei*, which are exclusively trunciflorous. Inflorescences occasionally form on new growth in the species with ramiflory (Fig. 1D). Six of the species have distributions centered in the Amazon Basin, while the other three are endemic to the Atlantic Forest. All are understory species. Petal configuration at anthesis is not always easy to observe in herbarium material but is particularly useful in separating species. There are three petal configurations: outer petals more or less erect, the inner petals squarrose (Figs 1I, 3B); petals of both whorls more or less erect (Fig. 1G); petals of both whorls spreading outward from the base, the apices sometimes curved slightly inward (Fig. 1C, E). Species with the first configuration belong to the XY-peruviana clade, while those of the latter two belong to the XY-aromatica subclade. Table 1 and the following key provide aids to the identification of trunciflorous *Xylopia* species.

**Table 1.** Comparison of cauliflorous *Xylopia* species with respect to distribution, maximum tree height, cauliflory type, petal orientation, and monocarp traits. T = trunciflory, R = ramiflory, (L) = from leaf axils, + = monocarps torulose, − = monocarps not torulose, i.e. not indented between seeds. Data assembled from herbarium specimens except for *X. decorticans*, which is based on information in Lobão and Johnson (2007).

**Species**	**Distribution**	**Maximum height (m)**	**Cauliflory type**	**Petal orientation at anthesis Outer/Inner**	**Monocarps**			
					**Shape in cross-section**	**Torulose**	**Apex**	**Pubescence**
**XY-peruviana clade**								
*X. atlantica*	Atlantic Forest	12	T, R	Unknown/Unknown	Terete	−	Blunt	Sparse
*X. decorticans*	Atlantic Forest	11	T, R(L)	Erect/Squarrose	Flattened	+	Blunt	Sparse
*X. ochrantha*	Atlantic Forest	15	T, R	Erect/Squarrose	Terete	−	Blunt	Dense
*X. rubrolineata*	Amazon Basin	25	T, R(L)	Erect/Squarrose	Terete	+	Blunt, Obtuse beak	Sparse
**XY-aromatica subclade**								
*X. benthamii*	Amazon Basin + Guianas	23	T	Spreading/Spreading	Terete	−	Blunt	Glabrous
*X. crinita*	Amazon Basin + Guianas	10	T, R(L)	Spreading/Spreading	Flattened	−	Blunt	Dense, becoming glabrate
*X. cuspidata*	Amazon Basin	12	T, R(L)	Erect/Erect	Flattened	+	Acute beak	Dense
*X. longicuspis*	Amazon Basin	5	T, R	Erect/Erect	Flattened	+	Hooked beak	Dense
*X. ulei*	Amazon Basin	15	T	Spreading/Spreading	Terete	+	Obtuse beak	Sparse

### Key to trunciflorous *Xylopia* species

**Table:** 

1	Petioles 15–20 mm long	***X. atlantica***
–	Petioles < 9 mm long	**2**
2	Leaf base rounded to subcordate	**3**
–	Leaf base cuneate to broadly cuneate	**5**
3	Outer petals narrowly oblong, > 3× as long as wide (Fig. 1E); apex of mature monocarp rounded	***X. crinita***
–	Outer petals ovate-triangular, ca. 2× as long as wide (Fig. 1G); apex of mature monocarp narrowed to a straight or curved beak	**4**
4	Sepals > ^1^/_2_ connate, 3–6 mm long, acute at apex; twigs with appressed hairs 0.5–1 mm long	***X. cuspidata***
–	Sepals < ^1^/_2_ connate, 10–12 mm long, acuminate at apex; twigs with ± spreading hairs 1–2 mm long	***X. longicuspis***
5	Twigs reddish brown, bark forming conspicuous flakes (Fig. 1D)	***X. decorticans***
–	Twigs brown to light gray, bark smooth or only slightly loose	**6**
6	Twigs with erect hairs 1.5–2 mm long (Fig. 1A); sepals and petals glabrous	***X. benthamii***
–	Twigs with appressed to loosely appressed hairs < 1 mm long; sepals and petals sparsely to densely pubescent	**7**
7	Outer petals > 3× as long as wide; leaves subsessile, petioles 1.5–2 mm long, blades 5.7–8 cm long, long-acuminate with apex forming ca. ^1^/_4_ of length	***X. ulei***
–	Outer petals ca. 2× as long as wide; leaves distinctly petiolate, petioles 3–9 mm long, blades 6.9–17.3 cm long, acuminate apex forming ^1^/_5_ or less of length	**8**
8	Outer petals 5–13 mm long; monocarps glabrate, torulose, pale green with a bright red abaxial stripe *in vivo*, with stipes 5–12 mm long, 1.2–1.6 mm thick, (Figs 3C, 3D, 4G); Amazon Basin species	***X. rubrolineata***
–	Outer petals 14–25 mm long; monocarps velutinous, smooth or faintly indented between seeds, greenish and faintly tinged with red *in vivo*, with stipes 4–8 mm long, 2.5–3 mm thick (Fig. 1B); Atlantic Forest species	***X. ochrantha***

## Discussion

### *Xylopia rubrolineata*, relationships and variation

Based on the details of its morphology, *Xylopia rubrolineata* is inferred to belong to the XY-peruviana clade and is most similar to *X. ochrantha* (Appendix 1, Fig. 2; Johnson et al. 2025; Nge et al. 2026). The diversification of XY-peruviana was accompanied by a shift to drier subhumid habitats (Johnson et al. 2025: figs 8, 9), and its species share pseudoterminal buds protected by bud scales. Pseudoterminal buds, while occurring in other *Xylopia* species, appear in XY-peruviana to be a morphological adaptation for dormancy during dry periods. The crown age of the clade is estimated as ca. 7–8 Ma (Nge et al. 2026), during the Late Miocene (ca. 11.6–5.3 Ma), a period of increased cooling and drying in tropical South America, as well as geologic change leading to the formation of the modern Amazon Basin (Latrubesse et al. 2010; Kirschner and Hoorn 2020; Hoorn et al. 2023). The distribution of the clade is restricted to the Amazon Basin and the Atlantic Forest. Connections between these areas from the Late Miocene onward have been established in a range of organisms (e.g., Costa 2003; Nicholls et al. 2025). A phylogenetic analysis of the genus *Inga* Mill. (Fabaceae) suggests that the genus spread from west to east between these areas (Nicholls et al. 2025), while a study of *Adenocalymma* (Bignoniaceae) indicated spread from east to west (Fonseca et al. 2026).

Given its wide distribution (EOO > 1 million km^2^), it is not surprising that *Xylopia rubrolineata* exhibits regional variation. Known localities are limited to three small areas (total AOO 128 km^2^): the southwestern Amazon Basin, the lower Amazon and adjacent Jarí River drainage, and the Serra dos Carajás area southeast of the Amazon (Fig. 5). Plants in the southwestern Amazon are shrubs or small trees 2–10 m in height; in the two eastern areas, plants are commonly taller. Petal shape and size also appeared to vary slightly between areas (cf. Figs 3A, 4Hfor outer petals, Figs 3B, 4Ifor inner petals), but this pattern was based on a small sample size due to the limited number of mature flowers available for study. The geographic discontinuities may be an artifact of undercollecting. Thus there were no grounds for separating these populations as distinct species.

If the distribution is disjunct, however, differences likely resulted from vicariance from an earlier continuous distribution or possibly a founder effect following dispersal (Johnson et al. 2025). Either scenario is in accord with habitat reconstructions at the Last Glacial Maximum, which indicate a changing complex of wet and dry habitats across the Amazon Basin (Pennington et al. 2000: fig. 6; Bonaccorso et al. 2006).

### Cauliflory in *Xylopia*

Despite a pantropical distribution of ca. 220 species, trunciflory in *Xylopia* is rare, limited to nine South American species in the XY-peruviana clade and one subclade of XY-aromatica. Both clades arose over a period of ca. 4 million years beginning ca. 7–8 Ma in the Amazon Basin and Atlantic Forest (Table 1, Fig. 2; Johnson et al. 2025; Nge et al. 2026), when overall Amazonian plant diversity declined significantly (Hoorn et al. 2023). The cauliflorous species are phylogenetically intermixed with non-cauliflorous species in both clades. This is in keeping with the pattern found in other plant groups (Harrison et al. 2012; Martínez-Velarde et al. 2023; Fonseca et al. 2026), suggesting that the cauliflory trait is evolutionarily labile, perhaps due to small but heritable changes in gene expression analogous to the regulation of axillary thorn production in *Gleditsia* L. (Xiao et al. 2023).

Did evolutionary history, habitat, floral biology, or seed dispersal shape cauliflory in *Xylopia*? The evolutionary history of *Xylopia* did not lead to extant exclusively cauliflorous radiations. The most parsimonious hypotheses include evolution of the trait at ca. 7–8 Ma in the XY-aromatica subclade and at ca. 6 Ma in the XY-peruviana clade, followed in both cases by several losses or gains, but cauliflory could have arisen at any time from the arrival of the genus in South America ca. 11.6 Ma (Fig. 2; Johnson et al. 2025; Nge et al. 2026). A formal analysis is outside the scope of this study and is complicated by weak branch support at key nodes within these two clades (Fig. 2).

As has been observed for most cauliflorous taxa, the trunciflorous species of both clades of *Xylopia* occur exclusively in the forest understory (heights in Table 1), as do other members of these clades. The non-cauliflorous species of the larger XY-aromatica clade, however, occupy both the canopy and the understory. No other aspects of habitat were found to distinguish cauliflorous species: examination of a principal components analysis (PCA) of 19 bioclimatic variables for *Xylopia* from Johnson et al. (2025: fig. S5) indicated that the species of the XY-peruviana clade and the XY-aromatica subclade occupy complementary, non-overlapping regions extending over the four quadrants of the PCA space. Species of the XY-peruviana clade do occupy drier habitats (mean annual precipitation 1264–2104 mm) than those of the XY-aromatica subclade (1858–3550 mm) (Johnson et al. 2025: fig. 8), but the cauliflorous species are intermixed with non-cauliflorous species within each precipitation range.

Floral biology in the Annonaceae, including *Xylopia*, favors outcrossing by means of protogyny and other floral behaviors (summarized by Saunders 2012; Johnson and Murray 2018), including floral phenology, petal shape and movement (Fig. 1; Table 1), fragrance, thermogenesis, timing and progression of anthesis, and even floral reflectance patterns (Küchmeister et al. 1998; Jürgens et al. 2000; Saunders 2012, 2020; Liu et al. 2025). Floral biology data are limited, but unrelated cauliflorous *Xylopia* species show no convergence in response to cauliflory in floral traits, as is seen, for example, in bat-pollinated plants (Fleming et al. 2009) or flagelliflorous Annonaceae (Teichert et al. 2012; Martínez-Velarde et al. 2023). In fact, related cauliflorous and non-cauliflorous *Xylopia* species have very similar flowers and petal movements (Appendix 1, Table 1, pers. obs.).

A generalist small-beetle pollination system has been inferred as ancestral in Annonaceae (Saunders 2012) and operates in both cauliflorous and non-cauliflorous *Xylopia* species (Küchmeister et al. 1998; Jürgens et al. 2000; Ratnayake et al. 2007). Species of the small-beetle families Nitidulidae and Staphylinidae are the most frequently reported visitors, but other small beetles, thrips, and flies may be attracted as well (Küchmeister et al. 1998; Saunders 2012; Johnson and Murray 2018; Saunders 2020; Saravy et al. 2021a, 2021b). The only known exception to these small, weak-flying pollinators was a large scarab beetle documented visiting the non-cauliflorous *X. annoniflora* Pombo & Zartman (Fig. 2; Pombo et al. 2017), a species with an atypically large floral chamber. Scarabs are also known as pollinators in other Neotropical Annonaceae genera with large floral chambers (e.g., *Cymbopetalum* Benth. and *Porcelia* Ruiz & Pavón, Murray 1993; *Annona* L., Saravy et al. 2021a). In tropical trees with small-insect pollinators, pollen movement and gene flow have been shown to be limited to short distances (see Dick et al. 2008; Grant et al. 2019), and this is likely to be the case in *Xylopia*. Cauliflory may, however, increase floral visitation and fruit set: Warren et al. (1997) reported that numbers of potentially pollinating insects were greater near trunk versus canopy inflorescences in *Theobroma cacao* L. (small midges and flies) and *Brownea latifolia* Jacq. (euglossine bees). Fruit set in these species was correspondingly greater as well. Cauliflory may likewise enhance pollination and fruit set in *Xylopia*.

Variation in fruit and seed morphology is highly constrained in *Xylopia* (Stull et al. 2017); cauliflorous and non-cauliflorous sister species were found to offer the same attractants and rewards, with only small quantitative differences in other traits. A variety of resident and non-resident birds and occasionally mammals disperse *Xylopia* seeds throughout the pantropical distribution of the genus (Johnson et al. 2025). In South America, ants provide secondary dispersal in *X. aromatica* (Lam.) Mart. (Christianini and Oliveira 2010), *X. peruviana* (label of *Maas et al. 5953* (F, NY, U)), and *X. sericea* A.St.-Hil. (Falcon et al. 2024). In *X. decorticans*, a rare cauliflorous species, ground-feeding birds (Horst et al. 2026) may also be secondary dispersers.

Inflorescence placement has been shown to affect types and numbers of dispersers in other plant groups (Shanahan and Compton 2001; Harrison et al. 2012; Thiel et al. 2023). Foraging stratification is a widely recognized phenomenon in birds, with extensive literature (see Chmel et al. 2021). Notably, Thiel et al. (2023) found that the cauliflorous liana *Marcgravia longifolia* (Marcgraviaceae) produced comparable numbers of fruits in the canopy, mid-level, and low-level of the forest, but frugivore guilds were highly stratified, including between birds of the same genus. In addition, flight speed and distance were inferred to be significantly higher for canopy than for understory frugivores (based on mean Kipp’s Index, a measure based on wing measurements: high = speed, distance; low = maneuverability). This is in accord with shorter gut passage times and smaller home ranges of understory birds (Thiel et al. 2023). Thus, it is suggested that, in *Xylopia*, a lack of fruits in the canopy in cauliflorous species entailed a shift in disperser guilds to favor shorter-distance understory dispersal. This is in contrast to long-distance dispersal, including to many islands, which has contributed significantly to geographic expansion in the genus (Johnson et al. 2025).

Amazonian plant diversity, as measured in the pollen record, declined significantly in the Late Miocene (Hoorn et al. 2023). With ongoing extinctions and fluctuating environments in South America, unpredictable differences between sites in species or abundance of *Xylopia* pollinators (Saravy et al. 2021b) and dispersers (Tongkok et al. 2020), especially in the canopy, might lead to a lack of successful reproduction. However, given the lack of change in floral and fruit morphology accompanying cauliflory, the generalist classes of pollinators and dispersers, i.e., small, weak-flying insects and birds, were likely to have remained the same. In addition to the altered inflorescence position, perhaps better suited to available vectors, cauliflorous flower production is not tied to new vegetative shoot growth, offering the possibility of rapid shifts in phenology and numbers of flowers (Armstrong and Marsh 1997; Harrison et al. 2012). Such changes in pollination and dispersal dynamics in *Xylopia* would be expected to augment pollination to ensure fruit set while limiting longer-distance dispersal and enhancing local dispersal. Overall, reduced genetic variability in cauliflorous *Xylopia* populations would result.

We hypothesize that cauliflory in *Xylopia* arose as an evolutionary bet-hedging adaptation to ensure reproductive success, preventing extinction. The bet-hedging model of Yasui (2022), which included long-lived organisms with multiple reproductive events and defined evolutionary bet-hedging as “any strategy to increase the between-generational geometric mean fitness to avoid extinction of its controlling genotype against unpredictable environmental fluctuation,” is applicable here. Although a trade-off between mean fitness and variance often results (e.g., Philippi and Seger 1989; Childs et al. 2010; Gianella et al. 2021; Overton and Sharkey 2021), Yasui (2022) argued that suppression of between-generation variance does not always reduce arithmetic mean fitness and that, in any case, costs to arithmetic mean fitness are sometimes unobservable. He suggests that geometric mean fitness generally provides a better measure of bet-hedging.

Two bet-hedging adaptations with features comparable to those of cauliflory in *Xylopia* are serotiny, a response to an unpredictable fire regime (Lamont et al. 2020; Gianella et al. 2021), and “ovule oversupply,” a response to unpredictable pollination at high elevation (Arroyo et al. 2019). Serotiny has arisen intermittently over ca. 150 million years in unrelated plant families (Lamont et al. 2020: figs 12, 13; Gianella et al. 2021) and is sometimes lost in populations of serotinous species growing in areas of lower fire frequency. In *Xylopia*, cauliflory arose within a much shorter timeframe (Fig. 2), but shows a similar pattern of intermittent occurrence at the species level. Intermittent occurrence is a characteristic of the cauliflory trait in other plant groups as well and suggests conservative bet-hedging, in which a trait is easily gained or lost (Crowley et al. 2016: fig. 1). “Ovule oversupply” as a bet-hedging response to unpredictable pollination was proposed by Arroyo et al. (2019) to account for increasing ovule numbers with elevation in numerous unrelated Andean flowering plants. In *Xylopia*, unreliable reproductive success of canopy inflorescences may be ameliorated with cauliflory, by means of increased numbers of flowers produced below the canopy, more effective pollinator or disperser guilds, or altered timing of floral production to track local climate changes. The cauliflorous trait in *Xylopia* meets the following criteria for bet-hedging: evolution in an unpredictable environment, a single labile trait with intermittent occurrence, and perhaps potential costs such as production of extra inflorescences, reduced long-distance dispersal, or inbreeding outweighed by long-term reproductive success (Crowley et al. 2016; Gianella et al. 2021; Yasui 2022).

Arroyo et al. (2019), Wang et al. (2020), and Yamamoto and Yasui (2024) suggest methodologies that could be applied to experimental studies of *Xylopia*. These might include comparisons between cauliflorous + non-cauliflorous sister species (Fig. 2), i.e., *X. decorticans* + *X. lanceolata* R.E. Fr. (allopatric) and *X. crinita* + *X. excellens* R.E. Fr. (sympatric, Küchmeister et al. 1998), to address questions such as: Was cauliflory accompanied by increased or prolonged flower production, augmenting pollination success, with reduced long-distance dispersal as a trade-off? Has inbreeding occurred in cauliflorous *Xylopia* species, leading to reduced genetic diversity? Or in *X. benthamii* and *X. ulei*, species in which ramiflory has been lost, has a more specialized, stable system evolved? Finally, ecological variables might be manipulated experimentally (Wibaux et al. 2025) to examine present-day unpredictable environmental impacts on reproductive success, such as fire or edge effects (Rocha et al. 2025; Horst et al. 2026).

## Conclusion

*Xylopia rubrolineata* is distinct from *X. ochrantha*, with which it had previously been confused. On the basis of morphology, it is inferred to belong to the XY-peruviana clade. Pseudoterminal vegetative resting buds, which were found in all species of the clade, may provide an adaptation for dormancy during dry periods.

Cauliflory in *Xylopia* is suggested to have altered pollination and dispersal dynamics to augment pollination and ensure fruit set while limiting longer-distance dispersal and enhancing local dispersal. It is hypothesized to have arisen as a bet-hedging trait ensuring reproductive success during a period of rapid ecological change in South America during the Late Miocene that led to many plant extinctions.

## Supplementary Material

XML Treatment for Xylopia rubrolineata

## Appendix 1

Recognition characters for members of the XY-peruviana clade (**bold** for possible synapomorphies). Many of these features are visible in the figures provided in the paper.

- Shrubs to (rarely) medium-sized trees, small trees most common
- **Rough peeling reddish brown bark** on twigs, extreme in *X. decorticans*
- Pseudoterminal buds covered by 2–3 cataphylls
- Leaves typically medium-sized, but varying from small (*X. laevigata*) to very large (*X. atlantica*); cuneate base versus rounded base separates from *X. cuspidata* and *X. longicuspis*, which have similar petals
- Inflorescences 1–2-flowered, more when trunciflorous
- **Pedicels/sepals/petals drying brown, often with appressed brown hairs**
- **Flower buds broad (ovoid), usually blunt at the apex**
- Sepals relatively short (6 mm or less) and slightly connate at base
- **Outer petals ovate, thickened, erect at anthesis, usually yellow**, sometimes pink
- Inner petals smaller, more rhombic and acuminate, thickened, **squarrose at anthesis**, slightly concave at the base, yellow marked with red at base (petals pink in *X. decorticans*)
- Stigmas blunt, barely connivent
- Torus and floral chamber small
- Monocarps ca. 4–18 per fruit
- Monocarps often red and glabrous at maturity (densely pubescent in *X. ochrantha*), with seeds oblique or nearly parallel to the long axis
- Seeds 1–7 per monocarp, commonly one or two, in a single row, typical bilobed aril, sarcotesta lacking, ca. 8–12 mm long, 5–6 mm wide

**Included species**.

*Xylopia atlantica* Mello-Silva & J.C. Lopes (Mello-Silva and Lopes 2014)

*Xylopia decorticans* D.M. Johnson & Lobão (Lobão and Johnson 2007)

*Xylopia laevigata* (Mart.) R.E. Fr. (Fries 1930)

*Xylopia lanceolata* R.E. Fr. (Fries 1930)

*X. langsdorfiana* A. St.-Hil. & Tul. [not sampled for phylogeny in Nge et al. 2026] (Fries 1930)

*Xylopia ochrantha* Mart. (Fries 1930)

*Xylopia peruviana* R.E. Fr. (Fries 1930)

*Xylopia rubrolineata* D.M. Johnson & N.A. Murray [not sampled for phylogeny in Nge et al. 2026] (this study)

## Additional information

### Artificial Intelligence (AI) use

The authors accept full responsibility for the content of the manuscript, including the disclosure of any use of AI.

No AI tools were used in the preparation of this manuscript.

### Author ORCIDs

M. Vaughn https://orcid.org/0009-0008-8498-8517

D. Harper https://orcid.org/0009-0006-7164-9829

N.A. Murray https://orcid.org/0000-0003-4076-6169

K. Floyd https://orcid.org/0009-0004-0546-3255

G.W. Stull https://orcid.org/0000-0002-2733-4823

D.M. Johnson https://orcid.org/0000-0003-2896-7419

### Data availability

All of the data that support the findings of this study are available in the main text or Supplementary Information.

## Supplementary materials

Supplementary material 1 *Xylopia ochrantha* KML file Data type kml Explanation note Occurences of *Xylopia ochrantha* from herbarium specimen labels and literature. https://binary.pensoft.net/file/1709960This dataset is made available under the Open Database License (http://opendatacommons.org/licenses/odbl/1.0/). The Open Database License (ODbL) is a license agreement intended to allow users to freely share, modify, and use this Dataset while maintaining this same freedom for others, provided that the original source and author(s) are credited. David M. Johnson, Keegan Floyd

Supplementary material 2 *Xylopia peruviana* KML file Data type kml Explanation note *Xylopia peruviana* occurences from herbarium specimen labels and literature. https://binary.pensoft.net/file/1709961This dataset is made available under the Open Database License (http://opendatacommons.org/licenses/odbl/1.0/). The Open Database License (ODbL) is a license agreement intended to allow users to freely share, modify, and use this Dataset while maintaining this same freedom for others, provided that the original source and author(s) are credited. David M. Johnson, Keegan Floyd

Supplementary material 3 *Xylopia rubrolineata* KML file Data type kml Explanation note *Xylopia rubrolineata* distribution occurrences from herbarium specimen labels. https://binary.pensoft.net/file/1709962This dataset is made available under the Open Database License (http://opendatacommons.org/licenses/odbl/1.0/). The Open Database License (ODbL) is a license agreement intended to allow users to freely share, modify, and use this Dataset while maintaining this same freedom for others, provided that the original source and author(s) are credited. David M. Johnson, Morgan Vaughn, Dekon Harper, Lexi Reiter, Elizabeth Dodds, Nancy A. Murray, Keegan Floyd

## References

[B1] Amancio G, Martínez-Velarde MF, Ortiz-Rodriguez AE (2026) Reproductive ecology in Mexican Annonaceae: what do we know? Botanical Sciences [in press]. 10.17129/botsci.3742

[B2] Armstrong JE, Marsh D (1997) Floral herbivory, floral phenology, visitation rate, and fruit set in *Anaxagorea crassipetala* (Annonaceae), a lowland rain forest tree of Costa Rica. The Journal of the Torrey Botanical Society 124: 228–235. 10.2307/2996610

[B3] Arroyo MTK, Pérez F, Jara-Arancio P, Pacheco D, Vidal P, Flores MF (2019) Ovule bet-hedging at high elevation in the South American Andes: evidence from a phylogenetically controlled multispecies study. Journal of Ecology 107: 668–683. 10.1111/1365-2745.13069

[B4] Bagstad K, Johnson DM (1999) Taxonomy of *Xylopia barbata* (Annonaceae) and related species from the Amazon/Orinoco region. Contributions from the University of Michigan Herbarium 22: 21–28.

[B5] Bazante ML, Alves M (2021) New records of Annonaceae in the [sic] Northeast Brazil. Acta Brasiliensis 5: 25–34. 10.22571/2526-4338449

[B6] Bonaccorso E, Koch I, Peterson AT (2006) Pleistocene fragmentation of Amazon species’ ranges. Diversity and Distributions 12: 157–164. 10.1111/j.1366-9516.2005.00212.x

[B7] Chatrou LW, Erkens RHJ, Richardson JE, Saunders RMK, Fay MF (2012) The natural history of Annonaceae. Botanical Journal of the Linnean Society 169: Frontispiece. 10.1111/j.1095-8339.2012.01242.x

[B8] Childs DZ, Metcalf CJE, Rees M (2010) Evolutionary bet-hedging in the real world: empirical evidence and challenges revealed by plants. Proceedings of the Royal Society B 277: 3055–3064. 10.1098/rspb.2010.0707 PMC2982066 20573624

[B9] Chmel K, Kamga SM, Awa T, Ewome FL, Uceda-Gómez G, Horák D, Mlíkovsky J, Molus LL, Riegert J, Janecek S (2021) Vertical stratification and seasonal changes of the avian community in Mount Cameroon lowland rainforest. African Journal of Ecology 00: 1–12. 10.1111/aje.12877

[B10] Christianini AV, Oliveira PS (2010) Birds and ants provide complementary seed dispersal in a neotropical savanna. Journal of Ecology 98: 573–582.

[B11] Costa LP (2003) The historical bridge between the Amazon and the Atlantic Forest of Brazil: a study of molecular phylogeography with small mammals. Journal of Biogeography 30: 71–86. 10.1111/j.1365-2745.2010.01653.x

[B12] Crowley PH, Ehlman SM, Korn E, Sih A (2016) Dealing with stochastic environmental variation in space and time: bet hedging by generalist, specialist, and diversified strategies. Theoretical Ecology 9: 149–161. 10.1007/s12080-015-0272-x

[B13] Dick CW, Hardy OJ, Jones FA, Petit RJ (2008) Spatial scales of pollen and seed-mediated gene flow in tropical rain forest trees. Tropical Plant Biology 1(1): 20–33. 10.1007/s12042-007-9006-6

[B14] Falcon JE, Schoereder JH, Ribeiro VS, Christianini AV, Camargo PH, Paolucci LN (2024) How do birds and ants contribute to the recruitment of a tropical tree? Biotropica 56: e13372. 10.1111/btp.13372

[B15] Fick SE, Hijmans RJ (2017) WorldClim 2: new 1-km spatial resolution climate surfaces for global land areas. International Journal of Climatology 37: 4302–4315. 10.1002/joc.5086

[B16] Fleming TH, Geiselman C, Kress WJ (2009) The evolution of bat pollination: a phylogenetic perspective. Annals of Botany 104: 1017–1043. 10.1093/aob/mcp197 PMC2766192 19789175

[B17] Fonseca LHM, Lohmann LG (2017) *Adenocalymma cauliflorum* (Bignonieae, Bignoniaceae), a new cauliflorous species from the Atlantic Forest of eastern Brazil. Systematic Botany 42: 584–589. 10.1600/036364417X696078

[B18] Fonseca LHM, Chatrou LW, Lohmann LG (2026) Atlantic Forest origins and Amazon connections: the evolutionary history of *Adenocalymma*. Annals of Botany [in press]. 10.1600/036364417X696078 PMC13454680 41973992

[B19] Fries RE (1930) Revision der Arten einiger Anonaceen-Gattungen. Acta Horti Bergiani 10: 1–128.

[B20] Gianella M, Bradford KJ, Guzzon F (2021) Ecological, (epi)genetic and physiological aspects of bet-hedging in angiosperms. Plant Reproduction 34(1): 21–36. 10.1007/s00497-020-00402-z PMC7902588 33449209

[B21] Gottstein M, Thiel S, Vornhagen JL, Mengel C, Tschapka M, Heymann EW, Heer K (2025) Gene flow and vertical stratification of pollination in the bat-pollinated liana *Marcgravia longifolia*. Ecology and Evolution 15: e72050. 10.1002/ece3.72050 PMC12374130 40860232

[B22] Grant EL, Conroy GC, Lamont RW, Reddell PW, Wallace HM, Ogbourne SM (2019) Short distance pollen dispersal and low genetic diversity in a subcanopy tropical rainforest tree, *Fontainea picrosperma* (Euphorbiaceae). Heredity 123(4): 503–516. 10.1038/s41437-019-0231-1 PMC6781113 31076650

[B23] Grant EL, Wallace HM, Brooks PR, Burwell C, Reddell PW, Ogbourne SM (2021) Floral attraction and flower visitors of a subcanopy, tropical rainforest tree, *Fontainea picrosperma*. Ecology and Evolution 11: 10468–10482. 10.1002/ece3.7850 PMC8328466 34367589

[B24] Harrison RD, Rønsted N, Xu L, Rasplus J, Cruaud A (2012) Evolution of fruit traits in *Ficus* subgenus *Sycomorus* (Moraceae): to what extent do frugivores determine seed dispersal mode? PLoS ONE 6: e38432. 10.1371/journal.pone.0038432 PMC3367955 22679505

[B25] Hoorn C, Lohmann LG, Boschman LM, Condamine FL (2023) Neogene history of the Amazonian flora: a perspective based on geological, palynological, and molecular phylogenetic data. Annual Review of Earth and Planetary Sciences 51: 419–446. 10.1146/annurev-earth-081522-090454

[B26] Horst MIA, Valadares RT, Calazans LSB, Bazante ML, Dutra VF (2026) A population census approach to conserving the threatened tree *Xylopia decorticans* (Annonaceae). Oryx First View: 1–6. 10.1017/S0030605325102524

[B27] IUCN (2012) IUCN Red List categories and criteria, Version 3.1, 2^nd^ Edn. Gland, Switzerland and Cambridge, UK, IUCN.

[B28] Johnson DM, Murray NA (2015) A contribution to the systematics of *Xylopia* (Annonaceae) in Southeast Asia. Gardens’ Bulletin Singapore 67: 361–386. 10.3850/S2382581215000307

[B29] Johnson DM, Murray NA (2018) A revision of *Xylopia* L. (Annonaceae): the species of tropical Africa. PhytoKeys 97: 1–252. 10.3897/phytokeys.97.20975 PMC10865103 38362585

[B30] Johnson DM, Murray NA (2020) A revision of *Xylopia* L. (Annonaceae): the species of Madagascar and the Mascarene islands. Adansonia, Sér. 3 42(1): 1–88. 10.5252/adansonia2020v42a1

[B31] Johnson DM, Nge FJ, Stull G, Murray NA, Floyd K, Streiff S, Rodrigues-Vaz C, Soulé VRC, Couvreur TLP (2025) Long-distance dispersals and ecological transitions underlie the biogeographic expansion of the pantropical magnoliid genus *Xylopia* (Annonaceae). Frontiers of Biogeography 18: e159992. 10.21425/fob.18.159992

[B32] Jürgens A, Webber AC, Gottsberger G (2000) Floral scent compounds of Amazonian Annonaceae species pollinated by small beetles and thrips. Phytochemistry 55: 551–558. 10.1016/S0031-9422(00)00241-7 11130664

[B33] Kirschner JA, Hoorn C (2020) The onset of grasses in the Amazon drainage basin, evidence from the fossil record. Frontiers of Biogeography 12: e44827. 10.21425/F5FBG44827

[B34] Küchmeister H, Webber AC, Silberbauer-Gottsberger I, Gottsberger G (1998) A polinização e sua relação com a termogênese em espécies de Arecaceae e Annonaceae da Amazônia Central. Acta Amazonica 28: 217–245. 10.1590/1809-43921998283245

[B35] Lamont BB, Pausas JG, He T, Witkowski ETF, Hanley ME (2020) Fire as a selective agent for both serotiny and nonserotiny over space and time. Critical Reviews in Plant Sciences 39(2): 140–172. 10.1080/07352689.2020.1768465

[B36] Latrubesse EM, Cozzuol M, da Silva-Caminha SAF, Rigsby CA, Absy ML, Jaramillo C (2010) The Late Miocene paleogeography of the Amazon Basin and the evolution of the Amazon River system. Earth-Science Reviews 99: 99–124. 10.1016/j.earscirev.2010.02.005

[B37] Liu M, Chen J, Pang C, Scharaschkin T, Saunders RMK (2025) More than fruity scents: pollination biology, scent, and spectral reflectance of Annonaceae species. Plant Species Biology 40: 403–425. 10.1111/1442-1984.70014

[B38] Lobão AQ, Johnson DM (2007) *Xylopia decorticans* (Annonaceae), a new cauliflorous species from Brazil. Contributions from the University of Michigan Herbarium 25: 207–211.

[B39] Maas PJM, Koek-Noorman J, Westra LYT (1993) Studies in Annonaceae. XVIII. New species from the Neotropics and miscellaneous notes. Botanische Jahrbücher für Systematik, Pflanzengeschichte und Pflanzengeographie 115: 77–95.

[B40] Martínez-Velarde MF, Rodrigues-Vaz C, Soulé VRC, Nge FJ, Schatz GE, Couvreur TLP, Ortiz-Rodriguez AE (2023) *Desmopsis terriflora*, an extraordinary new species of Annonaceae with flagelliflory. PhytoKeys 227: 181–198. 10.3897/phytokeys.227.102279 PMC10314296 37396012

[B41] Mello-Silva R, Lopes JC (2014) *Xylopia atlantica* (Annonaceae), new species from the coastal forest of Bahia, Brazil. Phytotaxa 188: 28–42. 10.11646/phytotaxa.188.1.5

[B42] Mildbraed J (1922) Wissenschaftliche Ergebnisse der zweiten Deutschen Zentral-Afrika-Expedition, 1910–1911, unter Fuhrung Adolf Friedrichs, Herzogs zu Mecklenburg. Band II: Botanik. Verlag von Klinkhardt & Biermann, 202 pp. [+ 61 plates]. https://www.biodiversitylibrary.org/item/15952

[B43] Murray NA (1993) Revision of *Cymbopetalum* and *Porcelia* (Annonaceae). Systematic Botany Monographs 40: 1–121. 10.2307/25027830

[B44] Nge FJ, Johnson DM, Murray NA, Holzmeyer L, Floyd K, Stull G, Soulé VRC, Sepulchre P, Tardif D, Rodrigues-Vaz C, Couvreur TLP (2026) Synchronous Miocene radiations and geographic-dependent diversification of pantropical *Xylopia* (Annonaceae). Molecular Phylogenetics and Evolution 215: 108485. 10.1016/j.ympev.2025.108485 41175901

[B45] Nicholls JA, Ringelberg JJ, Dexter KG, Loiseau O, Stone GN, Coley PD, Hughes CE, Kursar TA, Koenen EJM (2025) Continuous colonization of the Atlantic coastal rain forests of South America from Amazônia. Proceedings of the Royal Society B: Biological Sciences 292(2039): 1–13. 10.1098/rspb.2024.1559 PMC11750371 39837505

[B46] Onstein RE, Kissling WD, Chatrou LW, Couvreur TLP, Morlon H, Sauquet H (2019) Which frugivory-related traits facilitated historical long-distance dispersal in the custard apple family (Annonaceae)? Journal of Biogeography 46: 1874–1888. 10.1111/jbi.13552

[B47] Overton CE, Sharkey KJ (2021) Evolutionary bet-hedging in structured populations. Journal of Mathematical Biology 82. 10.1007/s00285-021-01597-z PMC8016807 33796960

[B48] Pennington RT, Prado DE, Pendry CA (2000) Neotropical seasonally dry forests and Quaternary vegetation changes. Journal of Biogeography 27: 261–273. 10.1046/j.1365-2699.2000.00397.x

[B49] Philippi T, Seger J (1989) Hedging one’s evolutionary bets, revisited. Trends in Ecology and Evolution 4: 41–44. 10.1016/0169-5347(89)90138-9 21227310

[B50] Pombo MM, Johnson DM, Chatrou LW, Zartman CE (2017) *Xylopia annoniflora* (Annonaceae): a new species from central Amazonia. Phytotaxa 317: 130–136. 10.11646/phytotaxa.317.2.5

[B51] Pontes-Pires AF, Barbosa MR de V, Johnson DM (2021a) *Xylopia maasiana* (Annonaceae), a new species from the Brazilian Amazon, and taxonomic notes on *Xylopia nitida* Dunal. Systematic Botany 46: 273–279. 10.1600/036364421X16231782047424

[B52] Pontes-Pires AF, Murray NA, Johnson DM, Barbosa MR de V (2021b) A new species of *Xylopia* (Annonaceae) from the Peruvian Amazon. Phytotaxa 514: 181–186. 10.11646/phytotaxa.514.2.11

[B53] Ratnayake RMCS, Gunatilleke IAUN, Wijesundara DSA, Saunders RMK (2007) Pollination ecology and breeding system of *Xylopia championii* (Annonaceae): curculionid beetle pollination, promoted by floral scents and elevated floral temperatures. International Journal of Plant Sciences 168: 1255–1268. 10.1086/521689

[B54] Richards PW (1952) The tropical rain forest. Cambridge University Press, 1–450.

[B55] Rocha LGd, Marimon Jr BH, Barradas AdC, Carvalho MACd, Soares CRA, Marimon BS, Ribeiro GHPdM, Oliveira EAd, Elias F, Emidio Jr C, Silva DRd, Garcia ML, Filho JAdR, Zortea M, Moreira ES, Domingues SCdO, Matricardi EAT, Galbraith D, Feldpausch TR, Oliveras I, Phillips OL (2025) Fire-induced floristic and structural degradation across a vegetation gradient in the southern Amazon. Forests 16: 1218. 10.3390/f16081218

[B56] Saravy FP, Marques MI, Schuchmann K (2021a) Coleopteran pollinators of Annonaceae in the Brazilian Cerrado—a review. Diversity 13: 438. 10.3390/d13090438

[B57] Saravy FP, Schuchmann KL, Marques MI (2021b) Diversity of insect flower visitors of *Xylopia aromatica* (Magnoliales, Annonaceae) in a Brazilian savanna. Diversity 13: 661. 10.3390/d13120661

[B58] Saunders RMK (2012) The diversity and evolution of pollination systems in Annonaceae. Botanical Journal of the Linnean Society 169: 222–244. 10.1111/j.1095-8339.2011.01208.x

[B59] Saunders RMK (2020) The evolution of key functional floral traits in the early divergent angiosperm family Annonaceae. Journal of Systematics and Evolution 58: 369–392. 10.1111/jse.12645 31639525

[B60] Shanahan M, Compton SG (2001) Vertical stratification of figs and fig-eaters in a Bornean lowland rain forest: how is the canopy different? Plant Ecology 153: 121–132. 10.1007/978-94-017-3606-0_10

[B61] Stull GW, Johnson DM, Murray NA, Couvreur TLP, Reeger JE, Roy CM (2017) Plastid and seed morphology data support a revised infrageneric classification and an African origin of the pantropical genus *Xylopia* (Annonaceae). Systematic Botany 42(2): 211–225. 10.1600/036364417X695484

[B62] Teichert H, Dötterl S, Frame D, Kirejtshuk A, Gottsberger G (2012) A novel pollination mode, saprocantharophily, in *Duguetia cadaverica* (Annonaceae): a stinkhorn (Phallales) flower mimic. Flora 207: 522–529. 10.1016/j.flora.2012.06.013

[B63] Thiel S, Willems F, Farwig N, Rehling F, Schabo DG, Schleuning M, Tello NS, Töpfer T, Tschapka M, Heymann EW, Heer K (2023) Vertically stratified frugivore community composition and interaction frequency in a liana fruiting across forest strata. Biotropica 55: 650–664. 10.1111/btp.13216

[B64] Thiers B (2026) [continuously updated] Index Herbariorum: A Global Directory of Public Herbaria and Associated Staff. https://sweetgum.nybg.org/ih/

[B65] Tongkok S, He X, Alcantara MJM, Saralamba C, Nathalang A, Chanthorn W, Brockelman WY, Lin L (2020) Composition of frugivores of *Baccaurea ramiflora* (Phyllanthaceae) and effects of environmental factors on frugivory in two tropical forests of China and Thailand. Global Ecology and Conservation 23: e01096. 10.1016/j.gecco.2020.e01096

[B66] Wallace AR (1878) Tropical nature and other essays. Macmillan & Co., 356 pp. 10.5962/bhl.title.69700

[B67] Wang XJ, Barrett SCH, Zhong L, Wu ZK, Li DZ, Wang H, Zhou W (2020) The genomic selfing syndrome accompanies the evolutionary breakdown of heterostyly. Molecular Biology and Evolution 38(1): 168–180. 10.1093/molbev/msaa199 PMC7782863 32761213

[B68] Warren JM, Emamdie DZ, Kalai (1997) Reproductive allocation and pollinator distributions in cauliflorus [sic] trees in Trinidad. Journal of Tropical Ecology 13: 337–345. 10.1017/S0266467400010543

[B69] Wibaux T, Lauri P-É, Kacou AAM, Kouakou OP, Vezy R (2025) A spatial perspective on flowering in cauliflorous cacao: architecture defines flower cushion location, not its early activity. Annals of Botany 136: 309–323. 10.1093/aob/mcaf107 PMC12445857 40492310

[B70] Xiao F, Zhao Y, Wang X, Sun Y (2023) Comparative transcriptome analysis of *Gleditsia sinensis* thorns at different stages of development. Plants (Basel) 12(7): 1456. 10.3390/plants12071456 PMC10096692 37050082

[B71] Yamamoto Y, Yasui Y (2024) Polyandry works as bet-hedging in the field cricket *Gryllus bimaculatus*, even after eliminating females in poor condition that cannot accept remating. Journal of Ethology 42: 61–69. 10.1007/s10164-023-00803-3

[B72] Yasui Y (2022) Evolutionary bet-hedging reconsidered: what is the mean–variance trade-off of fitness? Ecological Research 37(3): 406–420. 10.1111/1440-1703.12303

